# *Cathepsin K*-Cre Causes Unexpected Germline Deletion of Genes in Mice

**DOI:** 10.1371/journal.pone.0042005

**Published:** 2012-07-31

**Authors:** Crystal L. Winkeler, Raleigh D. Kladney, Leonard B. Maggi, Jason D. Weber

**Affiliations:** From the BRIGHT Institute and Department of Internal Medicine, Division of Molecular Oncology, Washington University School of Medicine, Saint Louis, Missouri, United States of America; Huntsman Cancer Institute, University of Utah, United States of America

## Abstract

Osteoclasts are terminally differentiated cells that attach to bone and secrete proteases to degrade the bone matrix. The primary protease responsible for the degradation of the organic component of the bone matrix is Cathepsin K, which was largely thought to be unique to osteoclasts. Given its apparent selective expression in osteoclasts, the *Cathepsin K* promoter has been engineered to drive the expression of Cre recombinase in mice and has been the most relevant tool for generating osteoclast-specific gene loss. In an effort to understand the role of the ARF tumor suppressor in osteoclasts, we crossed *Arf ^fl/fl^* mice to *Ctsk^Cre/+^* mice, which unexpectedly resulted in the germline loss of *Arf.* We subsequently confirmed Cre activity in gametes by generating *Ctsk^Cre/+^; Rosa^+^* mice. These results raise significant concerns regarding *in vivo* bone phenotypes created using *Ctsk^Cre/+^* mice and warrant further investigation into the role of Cathepsin K in gametes as well as alternative tools for studying osteoclast-specific gene loss *in vivo*.

## Introduction

Osteoclasts are bone-resorbing cells derived from the monocyte/macrophage family of the hematopoietic lineage [1]. Osteoclasts work in conjunction with osteoblasts, which are the bone-forming cells, to maintain a steady state of bone turnover. Osteoclasts tightly attach to bone in order to create an acidic milieu, called the resorption lacunae, that is necessary for the degradation of both the inorganic and organic components of bone matrix. Within the resorption lacunae, osteoclasts secrete proteases that degrade the organic component of bone once the inorganic component is demineralized by the secreted acid. Cathepsin K is a lysosomal cysteine protease of the papain family and is the primary protease responsible for the degradation of type I collagen [2], [3], [4]. In both humans and mice, Cathepsin K is highly expressed in osteoclasts. However, in humans, Cathepsin K has also been detected in heart, liver, and lung tissues [5]. During mouse development, Cathepsin K mRNA expression is highest in musculoskeletal tissues including bone, cartilage, and skeletal muscle and is predominantly expressed in osteoclasts [6]. Given the apparent selective expression of Cathepsin K in osteoclasts, a knockin mouse was created using the endogenous germline *Cathepsin K (Ctsk)* promoter to drive the expression of Cre recombinase solely in osteoclasts. In partially replacing the *Ctsk* locus with *Cre*, one allele of *Ctsk* is lost; however, *Ctsk^+/−^* mice are capable of maintaining normal bone turnover [7]. Furthermore, expression of Cre mRNA in *Ctsk^Cre/+^* mice is thought to be unique to bone, with Cre activity reported only in osteoclasts [8]. This system is important to the field of bone biology as it is the most relevant tool for assessing osteoclast-specific gene alterations *in vivo*.

In our initial study, we wanted to assess the role of the ARF tumor suppressor in osteoclasts. ARF is one product of the *Cdkn2a* locus, a site of frequent mutations in human cancer. While *Arf* shares two of its three exons with *INK4a* at the *Cdkn2a* locus, it is read in an alternative reading frame, such that it bears no functional homology to that of INK4a [9]. Mice that are void of *Arf* (but retain wild-type *INK4a*) begin developing tumors (fibrosarcomas and lymphomas) as early as 8 weeks of age and, after one year, 80% of *Arf^−/−^* mice succumb to spontaneous tumor development [10]. Together, the human and mouse data overwhelmingly places ARF as a key regulator of tumorigenesis and warrants an intensive characterization of ARF’s functions. We have previously shown that basal ARF suppresses protein synthesis in mitotic cells [11]. To further characterize the mitotic-independent growth suppressive functions of ARF, we wanted to know if ARF plays a role in the growth and function of non-dividing cells. To this end, we are characterizing the role of ARF in osteoclasts. To assess osteoclast-specific *Arf* loss *in vivo*, we crossed *Arf^fl/fl^* mice with *Ctsk^Cre/+^* mice. Here, we report the unanticipated finding that *Cathepsin K-*driven *Cre* expression causes germline deletion of floxed alleles in mice.

## Results

**Figure 1:**
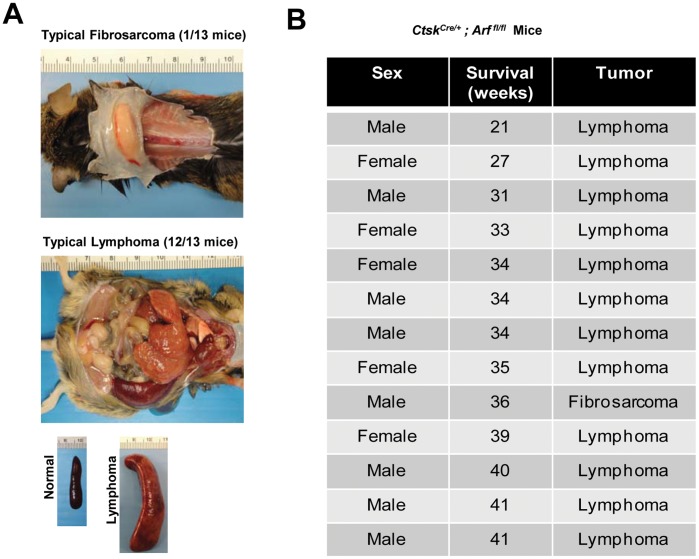
Crossing *Arf^fl/fl^* mice with *CtsK^Cre/+^* mice results in spontaneous tumor formation. (A) *Ctsk^Cre/+^*; *Arf^fl/fl^* mice display both fibrosarcomas (top image) and lymphomas with splenomegaly (middle and bottom images). (B) Spontaneous tumor development occurred in all *Ctsk^Cre/+^*; *Arf^fl/fl^* mice.

**Figure 2:**
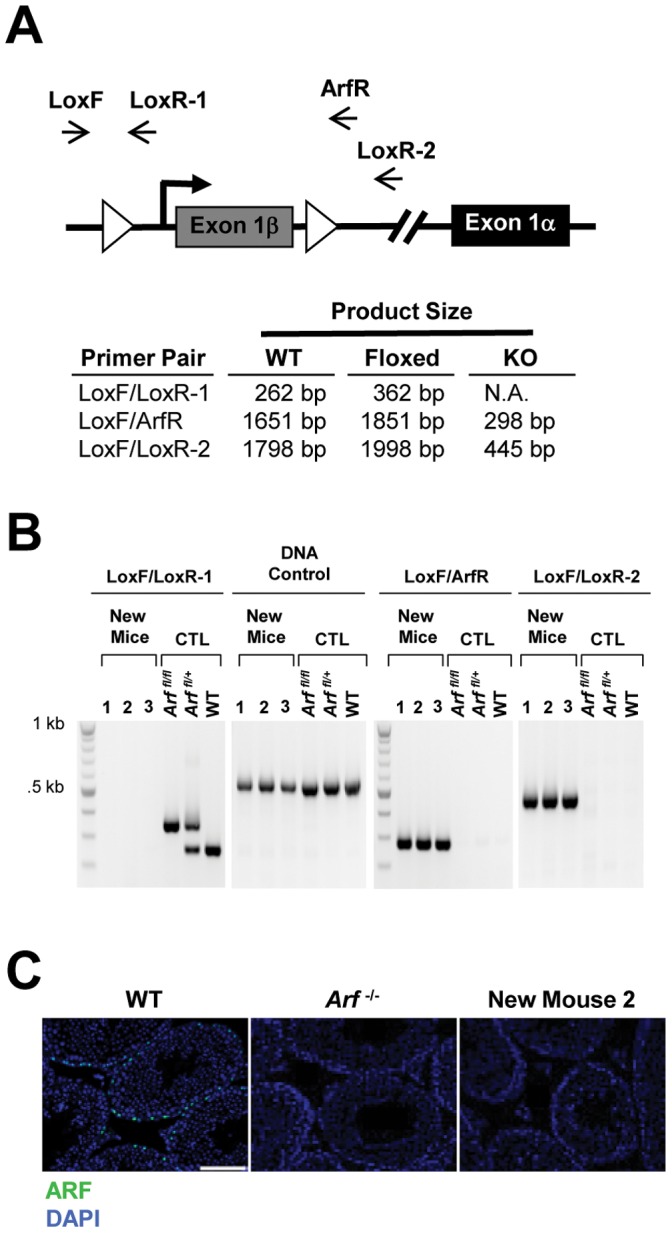
Crossing *Arf^ fl/fl^* mice with *CtsK^Cre/+^* mice results in germline *Arf* loss. (A) Three sets of primer pairs were designed to detect the presence of floxed *exon 1beta*, which is unique to *Arf.* (B) PCR products were not detected upon genotyping new mice for the presence of the 5′ loxP site (far left panel). This cannot be attributed to an absence of DNA (middle left panel). Controls are mice that were never crossed with *Ctsk^Cre/+^* mice. Two sets of primer pairs were used to detect the presence of *exon 1beta*. Each set detects a product indicating loss of *exon 1beta* (product size labeled as “KO” in table). (C) Immunofluorescent staining for ARF in testis tissues indicates the loss of *exon 1beta* at the protein level (scale bar = 100 µM).

To study osteoclast-specific *Arf* loss *in vivo*, we crossed *Ctsk^Cre/+^* mice with mice containing floxed *Cdkn2a exon1β*, which is unique to ARF [12]. Resultant litters of *Ctsk^Cre/+^; Arf ^fl/fl^* mice developed spontaneous fibrosarcomas and lymphomas with 100% penetrance (13/13 mice) (Fig. 1A,B), consistent with tumor formation observed in traditional *Arf ^−/−^* mice [10]. A primer set used to detect the presence of the 5′ loxP site (Fig. 2A, primer pair Lox F/Lox R-1) in tail DNA of *Arf^fl/fl^* mice was unable to amplify a product after the mice had been bred with *Ctsk^Cre/+^*, suggesting the loss of template (Fig. 2B, far left panel). We therefore reasoned that *exon1β* of *Arf* was not present in the tail DNA. To test this, we used two different primers sets that each contained a forward and reverse complementary sequence to a region of DNA outside of the loxP sites (Fig. 2A, primer pairs Lox F/Arf R and Lox F/Lox R-2). Both sets of primers resulted in products that suggested the loss of *exon1β* (Fig. 2B, right two panels). To further test for germline *Arf* loss, we exploited the fact that the testis is the only tissue in which we and others have been able to detect ARF protein by IHC [12]. Testes from mice, in which *exon1β* of *Arf* had seemingly been excised, were analyzed by IHC for ARF protein expression. While we detected ARF in the testis of wild-type mice, we were unable to detect ARF protein expression in mice showing loss of *exon1β* at the DNA level (Fig. 2C). Together, these results suggest that one copy of *Cre* under the control of the *Ctsk* promoter is sufficient to cause non-specific recombination and result in germline *Arf* loss.

**Figure 3:**
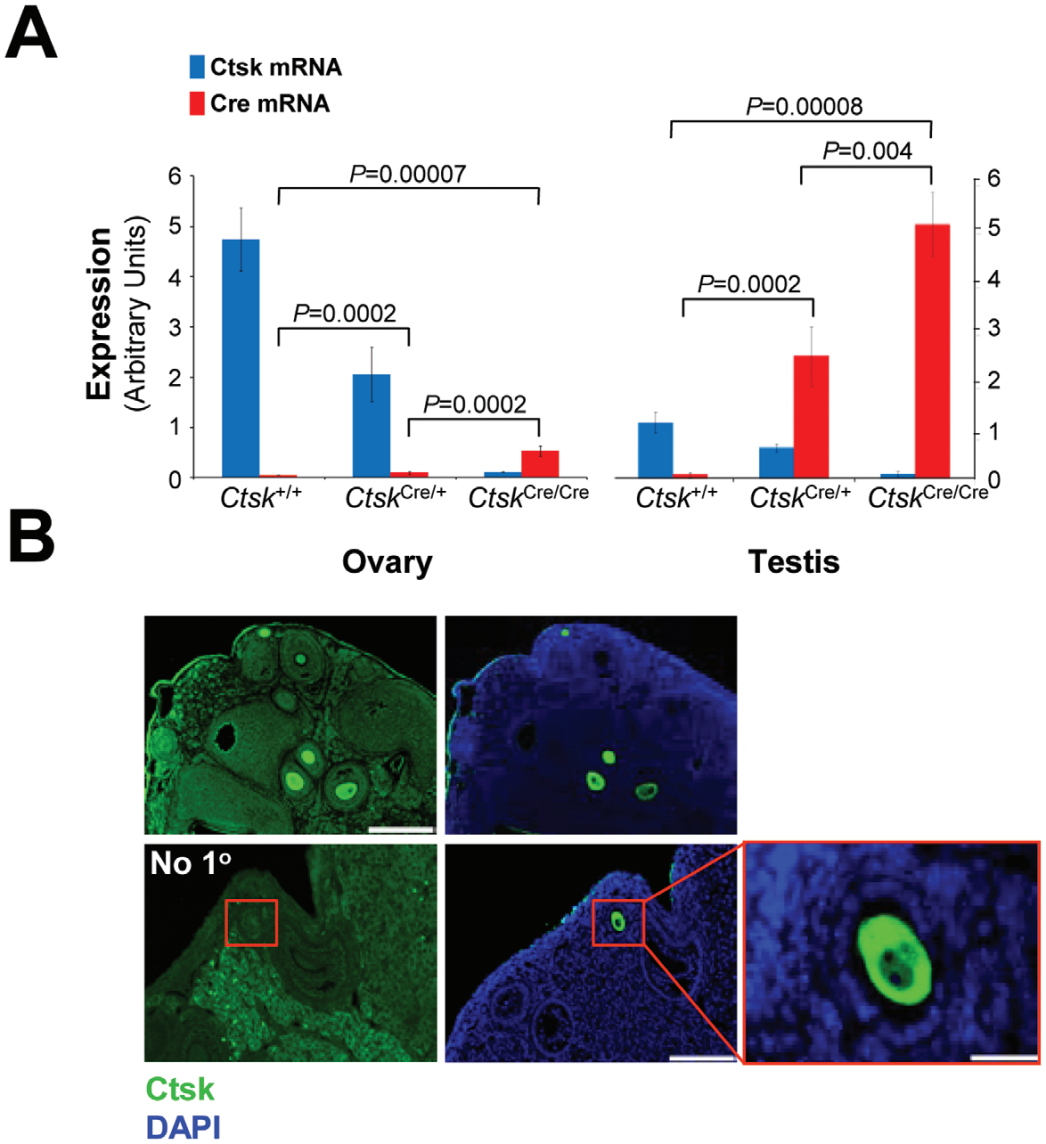
Cre is expressed in the gametes of *Ctsk^Cre/+^* mice. (A) Real-time PCR was used to quantify the presence of Cathepsin K mRNA (blue) and Cre mRNA (red) in ovary (left) and testis (right) for all indicated genotypes (n = 3). Data are represented as means ± SD. A two-tailed t-test was used to generate indicated p values. (B) Ovaries from WT mice analyzed by IHC for Cathepsin K. Top panels, scale bar = 200 µM. Bottom, left panel shows staining without primary antibody. An adjacent section to the control (bottom, middle) was incubated with primary antibody (scale bar = 100 µM). Red box indicates positively-stained oocyte (bottom, right scale bar = 20 µM).

**Figure 4:**
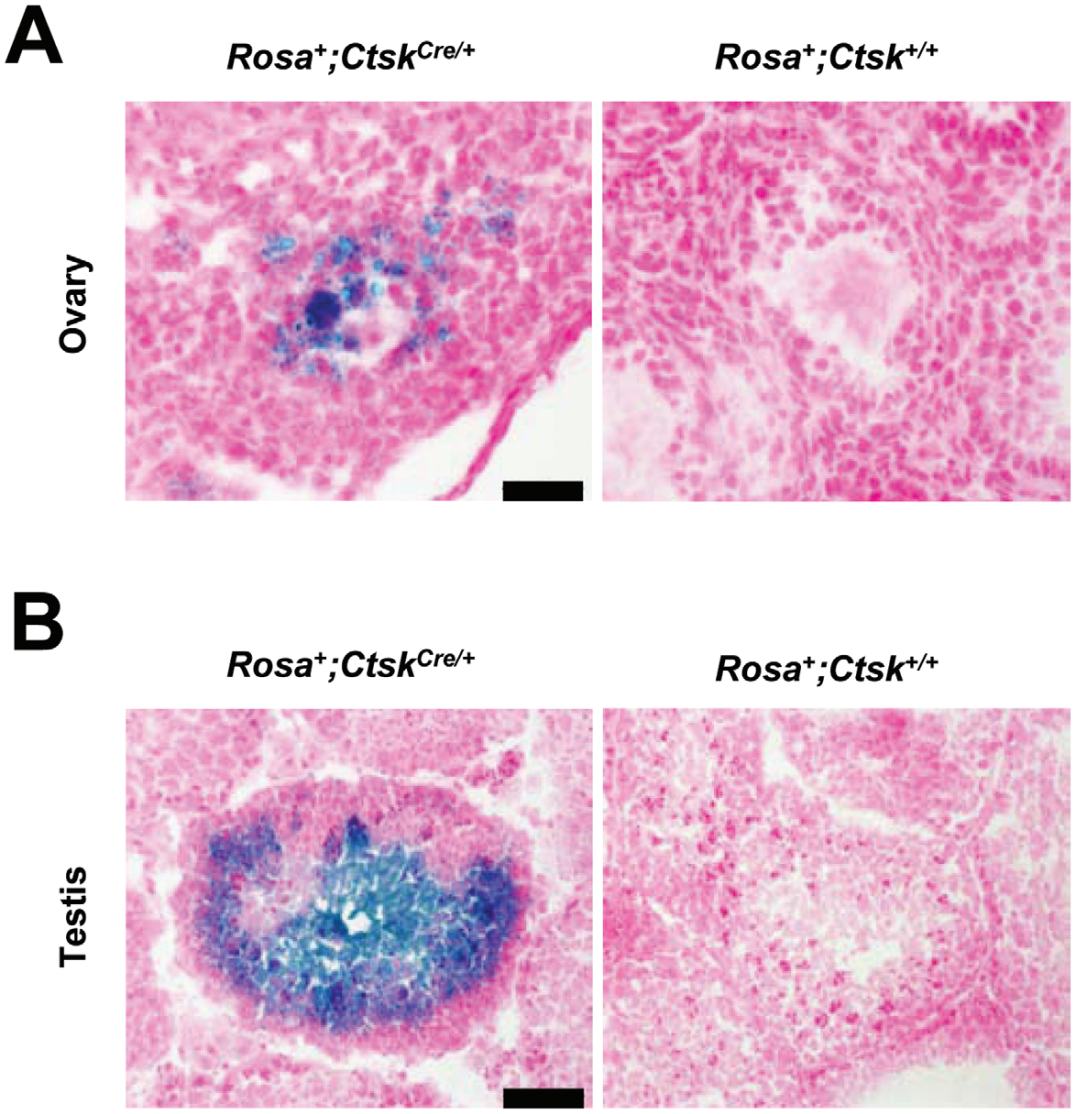
Cre expression in *Ctsk^Cre/+^* mouse gametes is verified with *Rosa^+^* mice. Reproductive organs of indicated genotypes were analyzed for Cre activity by LacZ staining. (A) In ovaries of *Rosa^+^*; *Ctsk^Cre/+^* mice, Cre activity was detected in oocytes and cells surrounding the developing oocytes (left panel). Controls were negative for LacZ staining (right panel). Scale bar = 50 µM. (B) In testes of *Rosa^+^*; *Ctsk^Cre/+^* mice, Cre activity was detected primarily in spermatozoa (left panel). Controls were negative for LacZ staining (right panel). Scale bar = 100 µM.

In light of our unexpected finding that *Cathepsin K*-driven Cre caused germline *Arf* loss when bred with *Arf ^fl/fl^* mice, we sought to assess the reproductive tissues of *Ctsk^Cre/+^* mice. We first investigated the presence of Cre and Cathepsin K mRNAs in ovary and testis tissues by qRT-PCR. In ovaries of 10-week-old wild-type mice, we detected Cathepsin K mRNA. Cathepsin K mRNA decreased as *Ctsk* was replaced by *Cre*. Accordingly, we detected increasing amounts of Cre mRNA as Cathepsin K mRNA decreased (Fig. 3A, left). Similar results for Cathepsin K mRNA were obtained using testes RNA from 8-week-old *wild-type* mice, albeit at much lower levels relative to that observed in ovaries. Importantly, we again detected a dose-dependent decrease in Cathepsin K mRNA as Cre mRNA levels increased in testes (Fig. 3A, right). Given the striking presence of Cathepsin K mRNA in ovaries, we analyzed ovary tissues from 16-week-old mice for the presence of Cathepsin K by immunohistochemistry (IHC). Our results indicate that Cathepsin K is expressed at the protein level in ovaries and is primarily localized to oocytes within the developing follicles (Fig. 3B). Furthermore, we noted that follicles expressing Cathepsin K were primarily those in the early stages of maturation. As a control, adjacent tissue sections were stained without primary antibody. Sections incubated without antibody were negative for Cathepsin K in positive areas of the adjacent section that was incubated with the primary antibody (Fig. 3B). These results indicate that Cathepsin K is present in mouse ovaries and may be expressed at a specific stage during follicle development. Finally, we wanted to confirm the presence of Cre in the reproductive tissues by assaying for recombinase activity. To confirm Cre activity, we crossed *Ctsk^Cre/+^* mice with *Rosa^+^* reporter mice. Ovary and testis tissues from *Rosa^+^; Ctsk^Cre/+^* mice were analyzed for Cre activity by LacZ staining. In ovaries, Cre activity was primarily detected in the oocyte and granulosa cells (Fig. 4A). In testes, abundant staining was evident in spermatozoa (Fig. 4B). This data demonstrates that *Cathepsin K*-driven Cre activity is present in gametes of both female and male mice. Of note, only one *Cre* allele was necessary for DNA excision at the *Rosa* locus. As a whole, this data demonstrates that crossing mice containing a floxed gene with *Ctsk^Cre/+^* mice can result in germ line loss of the floxed gene.

## Discussion

Together, our results demonstrate that Cathepsin K is expressed in gametes, and this expression can result in germline excision of a floxed allele when bred with *Ctsk^Cre/+^* mice. Within the ovary, we observed Cathepsin K protein expression primarily in oocytes. Data from Chiu *et al*. showing expression of Cathepsin K mRNA in mouse ovaries supports this finding [13]. Expression of Cathepsin K has also been noted in human ovary samples [14]. While there is no published role for Cathepsin K in ovaries, our data showing that Cathepsin K may be expressed during early stages of oocyte development suggests a possible role for Cathepsin K during oocyte maturation. Moreover, other Cathepsins have also been shown to be expressed during specific stages of follicle development in teleosts [15]. In testis, Cre activity was noted within seminiferous tubules and was largely localized to mature spermatozoa. To support this finding, when creating transgenic mice that express Cre driven by the *Ctsk* promoter, Chiu *et al.* published the expression of Cre expression in the testis of multiple mouse lines [13].

Upon crossing *Ctsk^Cre/+^* mice with *Arf^ fl/fl^* mice, we generated germline *Arf* loss as demonstrated by PCR, IHC for ARF in testis, and disease phenotypes that mimic traditional *Arf*-null mice [10]. Unfortunately, these findings place into question previous data that has been generated using mice containing *Cre* driven by the *Ctsk* promoter to create osteoclast-specific knockouts. Furthermore, our data may help explain the unexpected results that others have generated when using *Ctsk^Cre/+^* mice to generate osteoclast-specific gene ablation [16]. We are not suggesting that all animal models with *Cre* under the control the *Ctsk* promoter will result in a genomic knockout as neither every follicle nor every seminiferous tubule was positive for Cre activity. However, our data clearly indicate that careful testing is necessary when using this mouse model to ensure that the intended gene excision has occurred only in osteoclasts. Finally, these results warrant future investigation of an alternative tool to study osteoclast-specific gene loss *in vivo* and, in light of novel inhibitors intended to target Cathepsin K for therapy [17], a better understanding of the role of Cathepsin K in gametes.

## Materials and Methods

### Generation of Ctsk^Cre/+^; Rosa^+^ and Ctsk^Cre/+^; Arf ^fl/fl^ Mice

All animals were used in protocols that were reviewed and approved by the Washington University Animal Studies Committee. *Ctsk^Cre/+^* mice (generously provided by S. Kato) and the *Rosa* ^+^ reporter mice have been described previously [8], [18]. *Cathepsin K-Cre* “knockin” heterozygous mice (*Ctsk^Cre/^* ^+^; maintained on a C57BL/6 background) were crossed with mice heterozygous for an *R26R* allele, where a constitutively active chromosomal gene was manipulated to insert the *lacZ* gene such that the β-galactosidase protein is produced only following the removal of a “stuffer” fragment flanked by loxP sites (*Rosa^+^*; maintained on a C57BL/6 background). *Ctsk^Cre/+^* mice were also crossed with mice homozygous for a floxed allele of *Arf* where exon1β is flanked by loxP sites (*Arf ^fl/fl^*; maintained on a mixed background of C57BL/6 and 129SvJae, a kind gift from C. Sherr, St. Jude Children’s Research Hospital). *Ctsk* wild-type and “knockin” alleles were detected using primers Ctsk-P1 5′-TTATTCCTTCCGCCAGGATG-3′, Ctsk-P2 5′-TTGCTGTTATACTGCTTCTG-3′ and Ctsk-P3 5′-TAGTTTTTACTGCCAGACCG-3′. When used together in a PCR reaction, a wild-type allele generates a 135 bp fragment whereas a “knockin” allele produces a 300 bp fragment. *Arf* wild-type and floxed alleles were determined by using primers SpeLxF-30 5′-TTGCTACTTTACTGCAGCCAGACCACTAGG-3′ and SpeLxR-30 5′-CTCGGAGATTGAGAAAGCGGGAAGTCAAGC-3′ in which the wild-type allele generates a 260 bp product and the floxed allele generates a 360 bp product. The presence of the *R26R* allele was assessed by amplification of a 320 bp fragment using primers R26Rfwd 5′-AAAGTCGCTCTGAGTTGTTAT-3′ and R26Rrev 5′-GCGAAGAGTTTGTCCTCAACC-3′.

### Ethics

This study was carried out in strict accordance with the recommendations in the Guide for the Care and Use of Laboratory Animals of the National Institutes of Health. The protocol was approved by the Animal Studies Committee of Washington University (Permit Number: 20100268).

### Tissue Harvest and Histology

Testis and ovary tissues were taken from mice that were cardiac perfused with formalin, then fixed using a rapid microwave fixation. Fixed tissues were PBS washed then processed through graded alcohols and xylenes then embedded in paraffin. 5- µm tissue sections were immunostained. Alternatively, frozen testis and ovary tissues were prepared from sucrose perfused mice, embedded in OCT over liquid nitrogen, sectioned at 5- µm and LacZ stained.

### Immunostaining

All sections were deparaffinized, rehydrated, washed in PBS, and blocked with serum-free Protein block (Dako) for 30 min at room temperature. All immunostaining required antigen retrieval which was performed in a food steamer using a 1x Reveal decloaker buffer (pH6.0) (Biocare Medical). Antibodies for the following markers were diluted in Antibody diluent (Dako) and applied overnight at 4°C: rat anti-p19^ARF^ (1∶400, Abcam), and rabbit anti-Cathepsin K (1∶200, Abcam). A secondary antibody conjugated with Alexa Fluor 488 was placed on tissue sections for 1 hr at room temperature (1∶300, Invitrogen). Nuclei were counterstained using *Slow Fade* Gold Antifade reagent with 4′,6-diamidino-2-phenylindole (DAPI) (Invitrogen).

### Quantitative Real-time PCR

Ovary and testis tissues were isolated, flash-frozen in liquid nitrogen, and homogenized in RNA-Solv (Omega Bio-Tek). The SuperScript III first-strand synthesis system (Invitrogen) was used to generate first strand cDNA. Real-time PCR was performed with iQ SYBR Green Supermix (Bio-Rad) on an iCycler thermal cycler (Bio-Rad).

### LacZ Staining

Frozen sections were air-dried for 30 min at room temperature and fixed in 2% paraformaldehyde/0.125% glutaraldehyde in 1x PBS pH 7.4 for 5 min. Sections were washed sequentially in 2mM MgCl_2/_PBS, 2mM MgCl_2_, 0.02% Nonidet P40, 0.01% deoxycholate in PBS, and LacZ staining buffer (5 mM potassium ferrocyanide, 5 mM potassium ferricyanide, 2 mM MgCl_2,_ 1x PBS), and then incubated in LacZ staining buffer supplemented with 1 mg/ml X-Gal overnight at 37°C with shaking. Nuclei were counterstained with Nuclear Fast Red (Dako).

### Microscopy and Imaging

Gross pathological images were captured using a Sony Cyber-shot 12.1 megapixel digital camera equipped with a Carl Zeiss 5x optical zoom wide (28 mm) lens. Microscopy images for histology were obtained with a BX61 microscope (Olympus America) using the following objectives: UPlan Apochromatic 20X/NA 0.70, and UPlan Apochromatic 40X/NA 0.85. Tissue sections stained with LacZ were mounted with Krystalon (EMD). Histological microscopy images were obtained with a DP70 color Bayer mosaic digital camera, Peltier device cooled to −10°C (Olympus America). These images were captured with MicroSuite Biological Suite version 5 software (Olympus Soft Imaging Solutions) and resized and formatted with Adobe Photoshop CS3 software (Adobe Systems Incorporated). Fluorescence microscopy images were obtained with an Eclipse 90i microscope (Nikon) using the following objectives: Plan Apochromatic 10x/NA 0.45, Plan Apochromatic 20x/NA 0.75, and Plan Apochromatic Oil 100x/NA 1.40. Tissue sections for fluorescence microscopy images were mounted with *SlowFade* Gold Antifade reagent with DAPI (Invitrogen). Images were obtained using a CoolSnap HQ^2^ monochrome digital camera, Peltier cooled to −30°C (Photometrics). Fluorescence images were captured with MetaMorph version 7.6 software (MDS Analytical Technologies) and resized and formatted with Adobe Photoshop CS3 software.

### Statistical Analysis

Data were analyzed using Excel (Microsoft Office). Results are expressed as the mean ±SD, and statistical significance was determined using a two-tailed t-test.
